# Genome-Wide Identification of the R2R3-MYB Gene Family in *Solanum americanum* and Functional Analysis of Its Role in Fruit Coloration

**DOI:** 10.3390/biology15171480

**Published:** 2026-09-01

**Authors:** Yanbo Yang, Zhiying Gong, Yanwen Wang, Hang Li, Jiahong Li, Qihang Cai, Zuming Luo, Zhenghai Sun, Liping Li

**Affiliations:** 1 College of Landscape and Horticulture, Southwest Forestry University, Kunming 650224, China; yangyanbo225@163.com (Y.Y.); gzhiying0807@163.com (Z.G.); 19143282712@163.com (Y.W.); 15096698328@163.com (H.L.); 15125764154@163.com (J.L.); caiqihang98@163.com (Q.C.); 14787321343@163.com (Z.L.); 2 College of Ecology and Environment (College of Wetlands), Southwest Forestry University, Kunming 650224, China

**Keywords:** *Solanum americanum*, anthocyanin biosynthesis, fruit coloration, genome-wide identification, virus-induced gene silencing

## Abstract

Anthocyanins are key secondary metabolites responsible for fruit coloration in plants, and their biosynthesis is largely regulated by R2R3-MYB transcription factors. However, the R2R3-MYB regulators controlling fruit anthocyanin accumulation in wild *Solanum* species remain poorly understood. Here, *Solanum americanum* was used to identify candidate R2R3-MYB genes associated with fruit coloration through genome-wide identification, phylogenetic analysis, synteny analysis, expression profiling, and virus-induced gene silencing (VIGS). A total of 122 SaMYB genes were identified, and phylogenetic analysis revealed that SaMYB proteins clustered with *Arabidopsis thaliana* R2R3-MYB members in conserved subgroups, suggesting evolutionary conservation of this family. Synteny analysis identified 37 syntenic gene pairs among SaMYB genes, and the Ka/Ks values of all analyzable gene pairs were below 1, indicating that these duplicated genes are subject to functional constraint. Integrated analysis of phylogenetic relationships, protein structures, promoter cis-elements, and fruit developmental expression patterns identified *SaMYB59* and *SaMYB106* as candidate regulators of anthocyanin accumulation. VIGS analysis demonstrated that silencing *SaMYB106* reduced purple coloration, decreased anthocyanin content, and downregulated the expression of the structural gene *DFR*. These results indicate that *SaMYB106* functions as a positive regulator of fruit anthocyanin accumulation in *S. americanum*. This study provides insights into the molecular basis of fruit coloration in wild *Solanum* species.

## Simple Summary

Fruit color is an important trait for understanding and utilizing plants. Anthocyanins are natural pigments that give many plant fruits their red, purple, and other colors. However, the key genes that control fruit color formation remain poorly understood in many wild plants. *Solanum americanum* is a wild species of the genus *Solanum* with potential nutritional and medicinal value. Its mature fruits show a distinct purple color, but the genetic basis underlying fruit coloration remains unclear. In this study, we analyzed genes potentially associated with fruit color formation in *S. americanum* and identified *SaMYB106* as a candidate gene associated with purple fruit coloration. The results showed that reduced *SaMYB106* expression weakened the purple color of *S. americanum* fruits and decreased anthocyanin content, suggesting that this gene may promote anthocyanin production in fruits. This study improves our understanding of fruit color formation in wild *Solanum* plants and provides a basis for identifying and utilizing genes related to fruit color.

## 1. Introduction

Fruit color is a key plant trait associated with survival, reproduction, and environmental adaptation. As a visual signal, fruit color attracts animals and promotes seed dispersal, thereby increasing reproductive success and population persistence [1]. Therefore, fruit color provides an important trait for investigating plant adaptation and evolutionary processes under natural selection [2]. In natural ecosystems, fruit color also reflects plant adaptations to changing environmental conditions. For example, pigments such as anthocyanins can protect plants from ultraviolet radiation and improve their tolerance to high-light and other stresses [3]. Moreover, plants can adjust fruit coloration under fluctuating temperatures by modulating the activities of pigment biosynthetic enzymes [4]. Notably, fruit color is also closely associated with nutritional quality. Pigments such as anthocyanins act as natural antioxidants, and their accumulation contributes to fruit nutritional value [5,6,7]. In agriculture and horticulture, fruit color is an important breeding trait that influences consumer preference and crop economic value [8].

Fruit color formation mainly depends on the synthesis and accumulation of pigments, among which anthocyanins are key secondary metabolites responsible for red, purple, and dark coloration [3,9]. Anthocyanins are flavonoid compounds, and their biosynthetic pathway is highly conserved. This pathway originates from the phenylpropanoid pathway and involves a series of structural genes encoding key enzymes, including chalcone synthase (CHS), dihydroflavonol 4-reductase (DFR), anthocyanidin synthase (ANS), and UDP-glucose: flavonoid 3-O-glucosyltransferase (UFGT) [10,11,12]. The spatial and temporal expression patterns of these structural genes influence anthocyanin accumulation during fruit development and across different tissues, thereby affecting fruit color changes [13,14]. Anthocyanin accumulation is regulated by developmental stages, tissue types, and environmental factors [15]. During fruit development and ripening, anthocyanins usually accumulate rapidly at specific stages, showing distinct developmental and tissue-specific patterns [16]. Tissue-specific anthocyanin accumulation and environmental responses are mainly controlled by precise transcriptional regulation [10,13]. Therefore, systematic analysis of the transcriptional control of anthocyanin biosynthesis-related genes is essential for understanding fruit color formation and development.

In higher plants, anthocyanin biosynthesis is mainly regulated by the MYB–bHLH–WD40 (MBW) complex, a core transcriptional module controlling the anthocyanin biosynthetic pathway [10,13]. Within this complex, WD40 proteins mainly act as structural scaffolds, bHLH transcription factors influence transcriptional activation efficiency, and MYB transcription factors confer regulatory specificity by recognizing target gene promoters [14,17,18]. Among MYB members involved in anthocyanin regulation, R2R3-MYB transcription factors represent the largest group and exhibit extensive functional diversification, making them major regulators of anthocyanin biosynthesis [19]. Previous studies have shown that various R2R3-MYB members regulate fruit or floral coloration, and their expression patterns are closely associated with anthocyanin accumulation during specific developmental stages and in different tissues [20,21]. In pepper (*Capsicum annuum*), *CaMYB5* expression is significantly increased during the late stage of fruit development and is closely associated with fruit coloration [22]. Similarly, in eggplant, the expression patterns of *SmMYB1* and *SmMYB2* are consistent with stage-specific anthocyanin accumulation during fruit development and regulate anthocyanin biosynthetic pathway activation through interactions with bHLH factors [23]. Functional studies have further demonstrated that R2R3-MYB transcription factors directly influence fruit coloration by regulating the expression of key structural genes in the anthocyanin biosynthetic pathway [24,25,26]. For example, in apple (*Malus domestica*), *MdMYBA* directly controls anthocyanin accumulation in the peel, and allelic variation results in significant differences in red peel coloration [27]. In strawberry, *FaMYB10L* interacts with *bHLH3* to activate the expression of the anthocyanin transport-related gene *FaRAP*, thereby promoting anthocyanin accumulation in fruits [28]. Therefore, identification of R2R3-MYB family members, together with developmental expression analysis and functional validation, are essential for discovering key MYB regulators involved in fruit anthocyanin accumulation and elucidating the mechanisms underlying fruit coloration. However, these studies have largely focused on cultivated species, and R2R3-MYB regulators in wild *Solanum* species remain uncharacterized.

*Solanum americanum* is an annual herbaceous plant belonging to the genus *Solanum* in the family Solanaceae and is widely distributed in tropical and subtropical regions. *S. americanum* exhibits strong tolerance to both abiotic and biotic stresses, making it a valuable wild genetic resource for Solanum crop improvement [29,30,31]. Its genome has recently been assembled, enabling systematic gene family analyses in this wild species for the first time [32]. Fruit is an important agronomic organ in Solanum plants, and its color is associated with environmental adaptation and directly influences fruit quality and utilization value [33]. As key regulators of anthocyanin biosynthesis, R2R3-MYB transcription factors play important roles in fruit color formation. Some members are also involved in plant stress responses, suggesting additional roles beyond fruit coloration regulation [34]. However, the molecular mechanisms underlying fruit color formation in *S. americanum* remain poorly understood, and the roles of its R2R3-MYB family in fruit coloration are still unclear. Therefore, identification of the R2R3-MYB family in *S. americanum*, together with developmental expression analysis and functional validation, will help elucidate the transcriptional mechanisms underlying fruit coloration.

## 2. Materials and Methods

### 2.1. Identification of R2R3-MYB Genes in S. americanum

The genome sequence and annotation data used in this study were downloaded from the *S. americanum* accession SP2271 dataset published by Lin et al. [32]. Protein sequences of *A. thaliana* R2R3-MYB (AtMYB) transcription factors were downloaded from TAIR (https://www.arabidopsis.org/, accessed on 1 March 2026) according to the classification reported by Dubos et al. [35]. The HMM profile of the MYB gene family (PF00249) was downloaded from the Pfam database (http://pfam.xfam.org/, accessed on 1 March 2026). Potential homologous sequences in the *S. americanum* genome were identified using BLASTp (v2.14.0) with an E-value threshold of ≤1 × 10^−10^. In parallel, HMMER (v3.3.2) was used to search the *S. americanum* proteome against the MYB domain profile PF00249 from Pfam with default parameters (E-value ≤ 1 × 10^−5^). Candidate proteins identified by BLASTp and HMMER were combined for further domain verification. Conserved domains of candidate proteins were analyzed using the NCBI CD-Search tool (https://www.ncbi.nlm.nih.gov/, accessed on 2 March 2026), and proteins containing complete R2 and R3 MYB domains were retained. Candidate MYB members were classified according to the number and arrangement of MYB domains. Genes containing two MYB repeats at the N-terminus were retained, whereas MYB-related proteins containing only one or three MYB repeats were excluded. Finally, the R2R3-MYB genes of *S. americanum* used for subsequent analyses were obtained. These genes were named *SaMYB1*-*SaMYB122* according to their chromosomal positions (Table S1). The physicochemical properties of SaMYB proteins were predicted using the ExPASy ProtParam tool (https://www.expasy.org/, accessed on 5 March 2026).

### 2.2. Phylogenetic Analysis of SaMYB Proteins

Multiple sequence alignment of full-length R2R3-MYB protein sequences from *S. americanum* and *A. thaliana* was performed using MEGA software (v11.0). Based on the alignment results, an unrooted phylogenetic tree was constructed using MEGA software with the Neighbor-Joining (NJ) method. Bootstrap analysis was performed with 1000 replicates. Based on the phylogenetic relationships between SaMYBs and *A. thaliana* R2R3-MYBs, *S. americanum* R2R3-MYB members were classified into different phylogenetic subgroups according to the subgroup classification criteria of *A. thaliana* R2R3-MYBs [35].

### 2.3. Protein Domain Analysis, Conserved-Motif Analysis, and Promoter Cis-Element Analysis of SaMYB

Protein domain and conserved-motif analyses were performed on the identified SaMYB protein sequences to characterize their structural features. Based on the conserved domain annotation results obtained from NCBI CD-Search, SaMYB proteins containing two complete MYB repeats were confirmed. Conserved motifs of SaMYB proteins were analyzed using MEME Suite (v5.5.5, accessed on 6 March 2026), with the maximum number of motifs set to 10 and other parameters maintained at default settings. Based on the *S. americanum* genome annotation file, the 2000-bp upstream promoter regions of each SaMYB gene were extracted for cis-element analysis. The cis-elements in the promoter regions were analyzed using the PlantCARE website (https://bioinformatics.psb.ugent.be/webtools/plantcare/html/, accessed on 6 March 2026). Combined with the phylogenetic grouping results of SaMYB, the domains, motif compositions, and cis-elements of SaMYB proteins were visualized using TBtools software.

### 2.4. Synteny Analysis of SaMYB Genes

Genome-wide synteny analysis and selection pressure analysis were performed to investigate the evolutionary history of the SaMYB gene family in *S. americanum*. Based on the protein sequences of all annotated protein-coding genes and the genome annotation file of *S. americanum*, genome-wide synteny analysis was performed within the *S. americanum* genome using the MCScanX plugin in TBtools software (v2.052). The syntenic relationships among SaMYB genes within the *S. americanum* genome were subsequently extracted from the genome-wide synteny results. Default parameters of MCScanX were applied for synteny detection. Syntenic gene pairs were visualized using the Advanced Circos plugin in TBtools. Ka/Ks values of SaMYB syntenic gene pairs were calculated using the Simple Ka/Ks Calculator plugin. Codon alignments were generated based on the coding sequences of each syntenic gene pair using the built-in alignment method of the plugin. Gaps were treated according to the default settings of the plugin, and no additional Ks filtering threshold was applied.

### 2.5. Plant Materials and Treatments

The *S. americanum* materials used in this study were collected from Kunming, Yunnan Province, China. The plants were self-pollinated for three consecutive generations to ensure a uniform genetic background, and mature seeds were stored at 4 °C in the laboratory. Plump seeds free of pests and diseases were selected as experimental materials. Before sowing, the seeds were surface-sterilized with 2% (*v*/*v*) sodium hypochlorite solution for 10 min to remove surface microorganisms. After sterilization, the seeds were rinsed three times with sterile distilled water to remove residual disinfectant. The sterilized seeds were soaked in sterile distilled water at 37 °C for 12 h to promote seed imbibition and germination. The treated seeds were placed on moist sterile filter paper for germination at 28 °C. After radicle emergence, uniformly germinated *S. americanum* seedlings were transplanted into plastic seedling pots containing nutrient soil (peat soil:vermiculite:perlite = 3:1:1, *v*/*v*/*v*) and grown under controlled conditions in a growth chamber. The growth conditions were set as follows: a 16 h/8 h light/dark photoperiod, a temperature of 25 ± 2 °C, and a relative humidity of 60–70%. Plants were watered regularly during growth to ensure normal development. When plants reached the flowering and fruiting stages, fruit samples were collected at different developmental stages according to fruit developmental and coloration characteristics. The collected samples were immediately frozen in liquid nitrogen and stored at −80 °C for subsequent RNA extraction and gene expression analysis.

### 2.6. RNA Extraction and qRT-PCR Analysis

To analyze gene expression patterns during fruit development in *S. americanum*, total RNA was extracted from fruit samples collected at different developmental stages. The fruit samples stored at −80 °C were ground into a fine powder in liquid nitrogen, and total RNA was extracted using the FastPure Plant Total RNA Isolation Kit (Vazyme Biotech Co., Ltd., Nanjing, China) according to the manufacturer’s instructions. The integrity of the extracted RNA was assessed on 1% agarose gel electrophoresis, and RNA concentration and purity were determined using a spectrophotometer. Samples with A_260_/A_280_ ratios between 1.8 and 2.1 were selected for subsequent experiments. Equal amounts of RNA were reverse transcribed into cDNA using the All-In-One 5X RT MasterMix Kit (Applied Biological Materials Inc., Shanghai, China). The resultant cDNA was diluted and used as the template for qRT-PCR. qRT-PCR was performed using ArtiCanCEO SYBR qPCR Mix (Tsingke Biotechnology Co., Ltd., Beijing, China). The Actin gene (sp2271chr05.14976) was used as the reference gene for qRT-PCR, and target gene expression levels were normalized accordingly. qRT-PCR primers for each gene were designed using the Primer-BLAST (https://www.ncbi.nlm.nih.gov/, accessed on 15 March 2026) function in the NCBI database. The amplification specificity of each primer pair was evaluated by agarose gel electrophoresis and melting curve analysis before quantitative analysis. Three biological replicates were used for each sample, and three technical replicates were performed for each reaction. Relative gene expression levels were calculated using the 2^−ΔΔCt^ method.

### 2.7. Virus-Induced Gene Silencing and Phenotypic Analysis

Based on the nucleotide sequences of target genes, the SGN VIGS Tool (https://vigs.solgenomics.net/, accessed on 20 March 2026) was used to predict target fragments for virus-induced gene silencing (VIGS). Specific amplification primers were designed using Primer3 software (v.4.1.0) (Table S2). The primers were synthesized by TsingKe Biological Technology Co., Ltd. (Beijing, China). PCR amplification was performed using Taq PCR StarMix (GenStar, Beijing, China) with an annealing temperature of 58 °C and an extension time of 30 s. The PCR products were purified using the StarPrep DNA Gel Extraction Kit (GenStar, Beijing, China). The pTRV2 vector was linearized by digestion with the KpnI restriction enzyme. Homologous recombination between the vector and gene fragment was performed using the ClonExpress II One Step Cloning Kit (Vazyme, Nanjing, China). The recombinant plasmids were transformed into *Escherichia coli* TOP10 competent cells using the heat shock method. Single colonies were selected and sequenced by TsingKe Biological Technology Co., Ltd. (Beijing, China). The plasmids with confirmed sequences were transformed into *Agrobacterium tumefaciens* GV3101 for subsequent experiments. *Agrobacterium* strains carrying pTRV2-target gene and pTRV1 vectors were cultured separately in LB liquid medium containing 50 mg/L kanamycin (Kan). The *Agrobacterium* cultures were grown at 28 °C and 180 rpm until the OD_600_ reached 0.8. The bacterial cells were resuspended to an OD_600_ of 1.0 using resuspension solution containing 2.3 g/L 1/2 MS, 20 g/L glucose, 1.95 g/L MES, and 100 μM acetosyringone (AS) at pH 5.8. A 1 mL syringe was used to infiltrate three pairs of mature leaves below the inflorescence of mature *S. americanum* plants. Three independent plants were used for each treatment. Fruits from three fruit clusters at the same developmental stage were analyzed for each plant, with 4–6 fruits per cluster. Fruit coloration phenotypes were observed at different days after infiltration. Anthocyanin contents were measured at 23 days after pollination, corresponding to the full coloration stage of wild-type fruits. Fruit anthocyanin content was measured with slight modifications based on a previous study [36,37]. A total of 0.3 g of fresh *S. americanum* fruit peel was weighed and extracted with 1 mL methanol containing 1% HCl. The samples were extracted overnight at 4 °C in darkness. The extracts were centrifuged at 12,000× *g* for 3 min, and the absorbance values of the supernatants at 530 nm and 657 nm were measured using a spectrophotometer. The total anthocyanin content was calculated using the formula: Q = (A_530_ − 0.25A_657_) × M^−1^. In this formula, Q represents the relative anthocyanin content, A_530_ and A_657_ represent the absorbance values measured at 530 nm and 657 nm, respectively, and M represents the fresh weight of the fruit peel sample (g). Three independent plants were used as biological replicates, and each biological replicate was measured with three technical replicates.

## 3. Results

### 3.1. Genome-Wide Identification of R2R3-MYB Members in S. americanum

**Figure 1:**
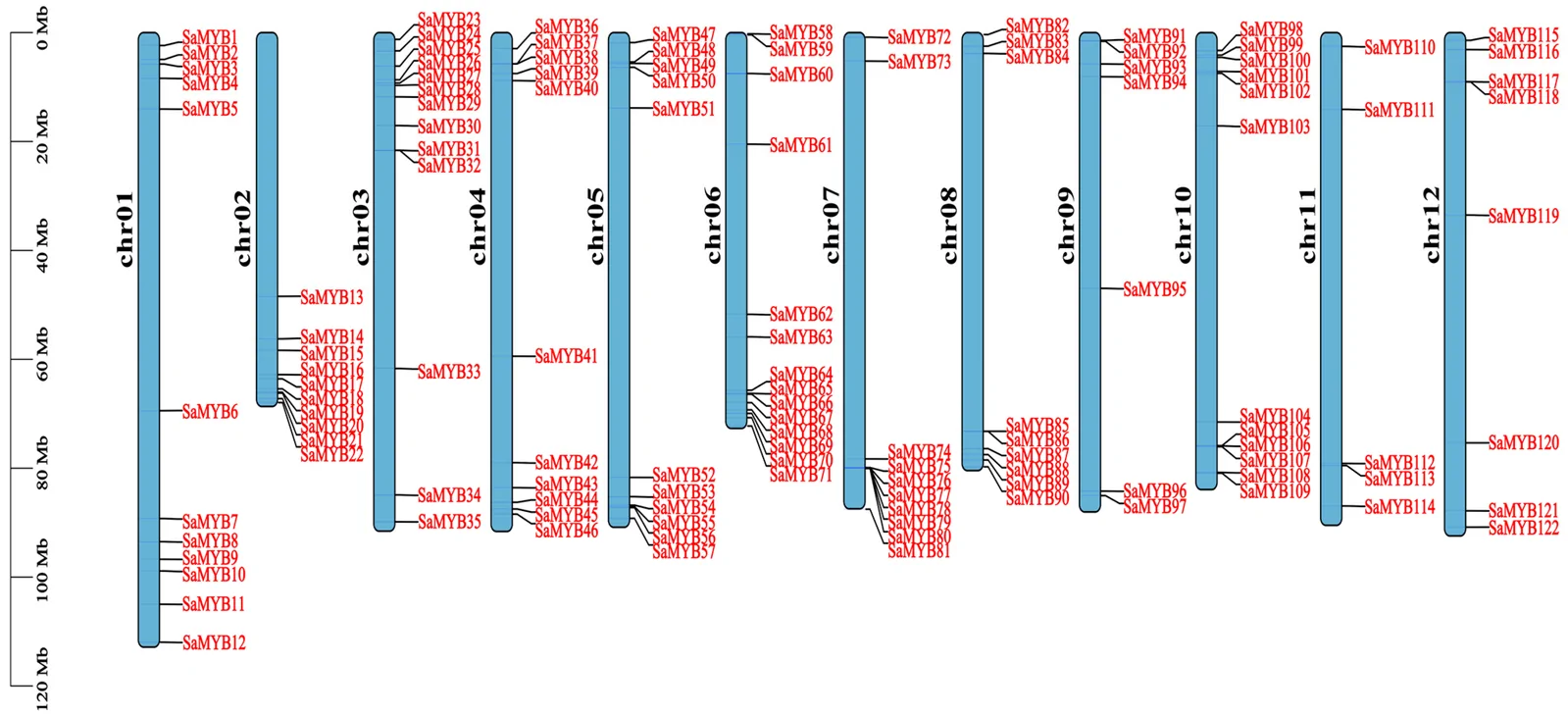
Chromosomal distribution of R2R3-MYB family members in *S. americanum*.

Based on the *S. americanum* genome sequence, a total of 122 R2R3-MYB genes were identified. Chromosome localization analysis showed that these genes were distributed across all 12 chromosomes of *S. americanum*, with no members located on unanchored scaffolds (Figure 1). The distribution of R2R3-MYB genes across chromosomes was uneven. These genes were relatively enriched on chr01, chr03, chr04, chr05, chr06, chr08, and chr10, whereas fewer members were located on chr09, chr11, and chr12. Based on their chromosomal positions, all R2R3-MYB genes were systematically named *SaMYB1*-*SaMYB122* (Table S1). Physicochemical property analysis showed that the 122 SaMYB proteins ranged from 216 to 601 amino acids in length, with molecular weights of 25.06–66.66 kDa and theoretical isoelectric points (pI) of 4.67–9.64. Most members exhibited neutral to slightly alkaline characteristics (pI approximately 5–9), and their GRAVY values were mostly negative, indicating that these proteins were generally hydrophilic (Table S3). The instability index analysis showed that the instability indices of SaMYB proteins ranged from 31.28 to 75.31. Most members had values above 40, suggesting that most SaMYB proteins were predicted to be unstable. These results indicated that SaMYB proteins possess diverse physicochemical characteristics.

### 3.2. Phylogenetic Analysis of SaMYB Members

**Figure 2:**
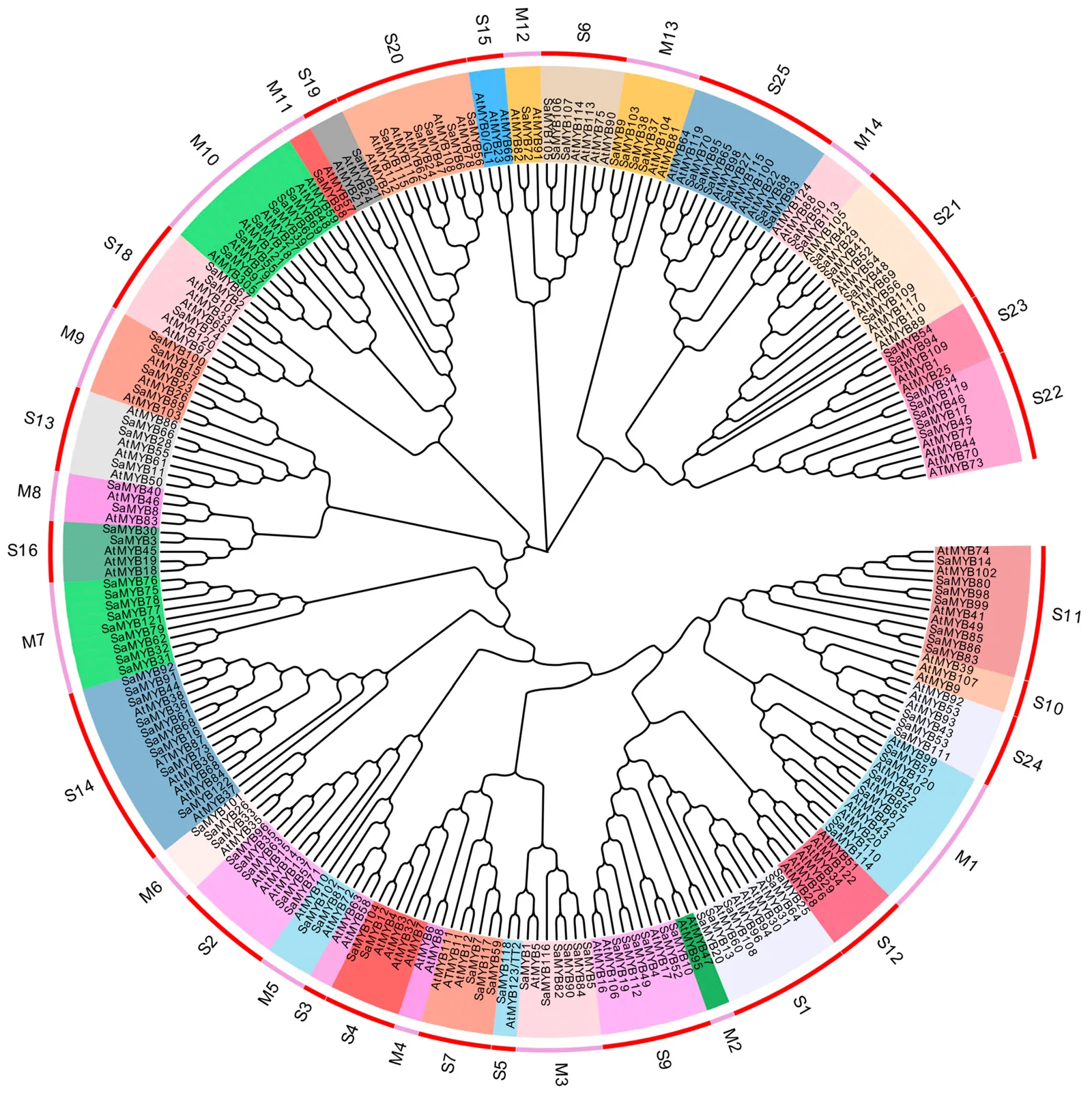
Phylogenetic tree of SaMYB proteins. Different colored blocks and the corresponding outer-ring labels represent distinct phylogenetic subgroups. SaMYB denotes R2R3-MYB family members from *S. americanum*, whereas AtMYB denotes R2R3-MYB family members from *A. thaliana*.

To investigate the phylogenetic relationships of the R2R3-MYB family in *S. americanum*, an unrooted phylogenetic tree was constructed using the protein sequences of SaMYBs and *A. thaliana* R2R3-MYBs (AtMYBs) (Figure 2, Supplementary File S1). Based on the classical classification system of *A. thaliana* R2R3-MYBs [35], SaMYB proteins were assigned to 25 known subgroups (S1–S25), while branches that could not be assigned to the established *A. thaliana* subgroups were designated as M1–M14. Phylogenetic analysis showed that most SaMYB proteins were clustered with AtMYB proteins within the same subgroups, suggesting that some R2R3-MYB members may share conserved evolutionary relationships between *S. americanum* and *A. thaliana*. Among these subgroups, the M7 subgroup contained nine SaMYB members without corresponding *A. thaliana* members, suggesting that these genes may represent lineage-specific diversification in *S. americanum*. Notably, several SaMYB members were distributed in the S4, S5, S6, and S7 subgroups. The S4 subgroup included *AtMYB3*, *AtMYB4*, *AtMYB7*, and *AtMYB32*, which are involved in the negative regulation of phenylpropanoid and flavonoid biosynthesis, thereby limiting substrate availability for anthocyanin biosynthesis [18,38,39]. The S5 subgroup included *AtMYB123*/*AtTT2*, which promotes proanthocyanidin biosynthesis by regulating downstream structural genes [35,40]. The S6 subgroup included *AtMYB75*/*PAP1*, *AtMYB90*/*PAP2*, *AtMYB113*, and *AtMYB114*, all of which positively regulate anthocyanin biosynthesis in *A. thaliana* by activating the expression of associated structural genes, thereby promoting anthocyanin accumulation and contributing to tissue pigmentation [18,38,39]. The S7 subgroup included *AtMYB11*, *AtMYB12*, and *AtMYB111*, which primarily promote flavonol biosynthesis and may affect metabolic flux within the anthocyanin biosynthetic pathway by regulating the flavonoid metabolic branch [41]. Therefore, SaMYB members in these subgroups may represent candidate regulatory factors associated with fruit coloration.

### 3.3. Synteny Analysis of SaMYB Genes

**Figure 3:**
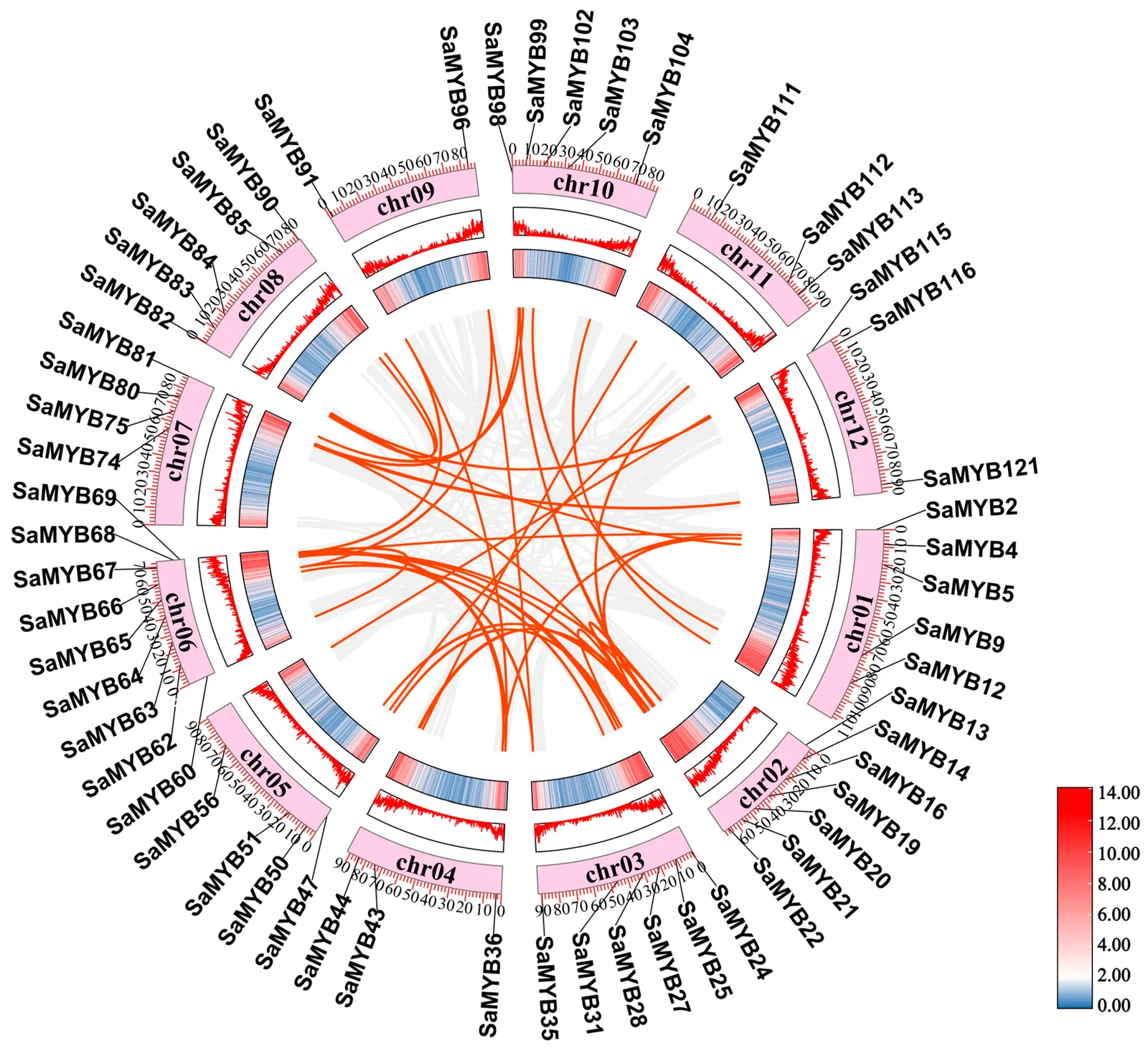
Syntenic relationships among SaMYB genes. Gray lines indicate genome-wide syntenic gene pairs, whereas red lines indicate syntenic gene pairs among SaMYB genes. The outer bars represent chromosomes, and the color gradient within each bar indicates gene density along the chromosomes.

Synteny analysis was conducted to investigate the evolutionary relationships and duplication patterns of SaMYB genes in the *S. americanum* genome (Figure 3). A total of 37 syntenic gene pairs were identified among SaMYB genes across different chromosomal regions. Several SaMYB genes showed syntenic relationships with multiple members. For example, *SaMYB25*, *SaMYB64*, and *SaMYB36* each formed syntenic gene pairs with multiple SaMYB members, indicating complex syntenic relationships within the SaMYB gene family. Ka/Ks values were subsequently calculated for the syntenic SaMYB gene pairs. Among the 36 gene pairs with available Ka/Ks values, all values were below 1, ranging from 0.056 to 0.536, indicating that these duplicated gene pairs were generally subject to functional constraints (Table S4).

### 3.4. Structural Characteristics, Motif Analysis, and Promoter Cis-Element Analysis of Anthocyanin Biosynthesis-Related SaMYB Members

**Figure 4:**
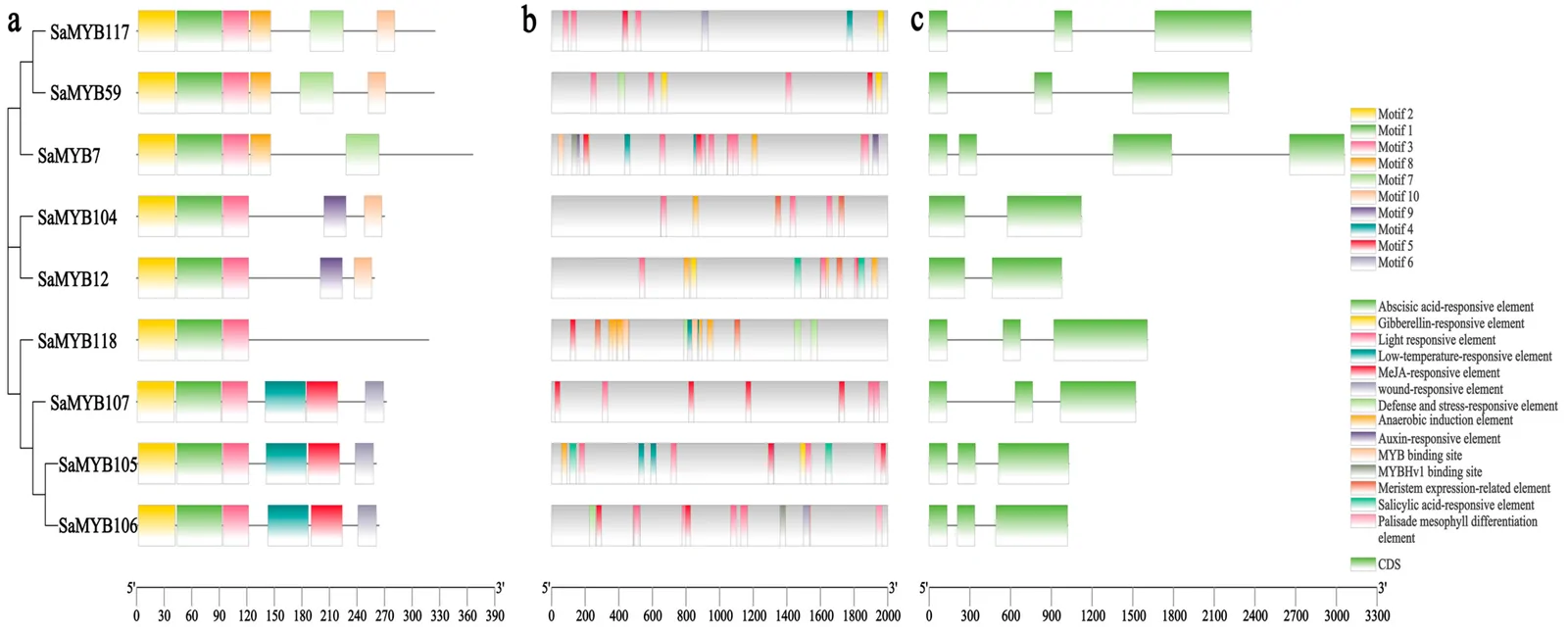
Gene structure, conserved motif, and promoter cis-acting element analyses of anthocyanin biosynthesis-related SaMYB genes. (**a**) Conserved motif analysis. Different colors represent different conserved motifs. (**b**) Promoter cis-acting element analysis. Different colors represent different types of cis-acting elements. (**c**) Gene structure analysis. Green boxes represent coding sequences (CDS).

Gene structures, conserved protein motifs, and promoter cis-elements of SaMYB genes in the S4, S5, S6, and S7 subgroups were analyzed to characterize SaMYB members potentially associated with anthocyanin biosynthesis (Figure 4). Exon-intron structure analysis showed that members within the same phylogenetic subgroup generally exhibited similar gene structures, whereas differences were observed among different subgroups. Members of the S4, S5, and S6 subgroups showed similar exon numbers and arrangement patterns, whereas the S7 subgroup displayed differences in intron length and structural complexity. MEME analysis identified 10 conserved motifs among the SaMYB proteins. Motifs 1, 2, and 3 were highly conserved among all SaMYB members and corresponded to the R2 and R3 MYB repeats. Members within the same subgroup exhibited similar motif compositions and arrangements, consistent with their phylogenetic classification. In contrast, differences in C-terminal motif compositions were observed among different subgroups. Cis-element analysis of the 2000 bp promoter regions showed that SaMYB genes contained diverse regulatory elements associated with light, hormone, stress, and developmental responses. The distribution patterns of these elements varied among subgroups, suggesting potential differences in transcriptional regulation among SaMYB members. S6 subgroup members, including *SaMYB105*, *SaMYB106*, and *SaMYB107*, contained multiple light- and hormone-responsive elements, particularly ABA- and MeJA-responsive elements, suggesting their potential regulation by developmental and environmental signals. S7 subgroup members also harbored light- and hormone-responsive elements, while *SaMYB7* additionally contained low-temperature-responsive and MYB-binding elements, suggesting a possible association with diverse regulatory pathways. In contrast, S4 and S5 subgroup members showed distinct promoter characteristics. *SaMYB118* contained multiple anaerobic induction, defense-related, and low-temperature-responsive elements, whereas *SaMYB104* and *SaMYB12* mainly contained light- and hormone-responsive elements. These differences in promoter composition suggest divergent regulatory patterns among SaMYB subgroups. However, the biological functions of individual cis-elements require further experimental validation.

### 3.5. Expression Patterns of SaMYB Genes During Fruit Coloration

**Figure 5:**
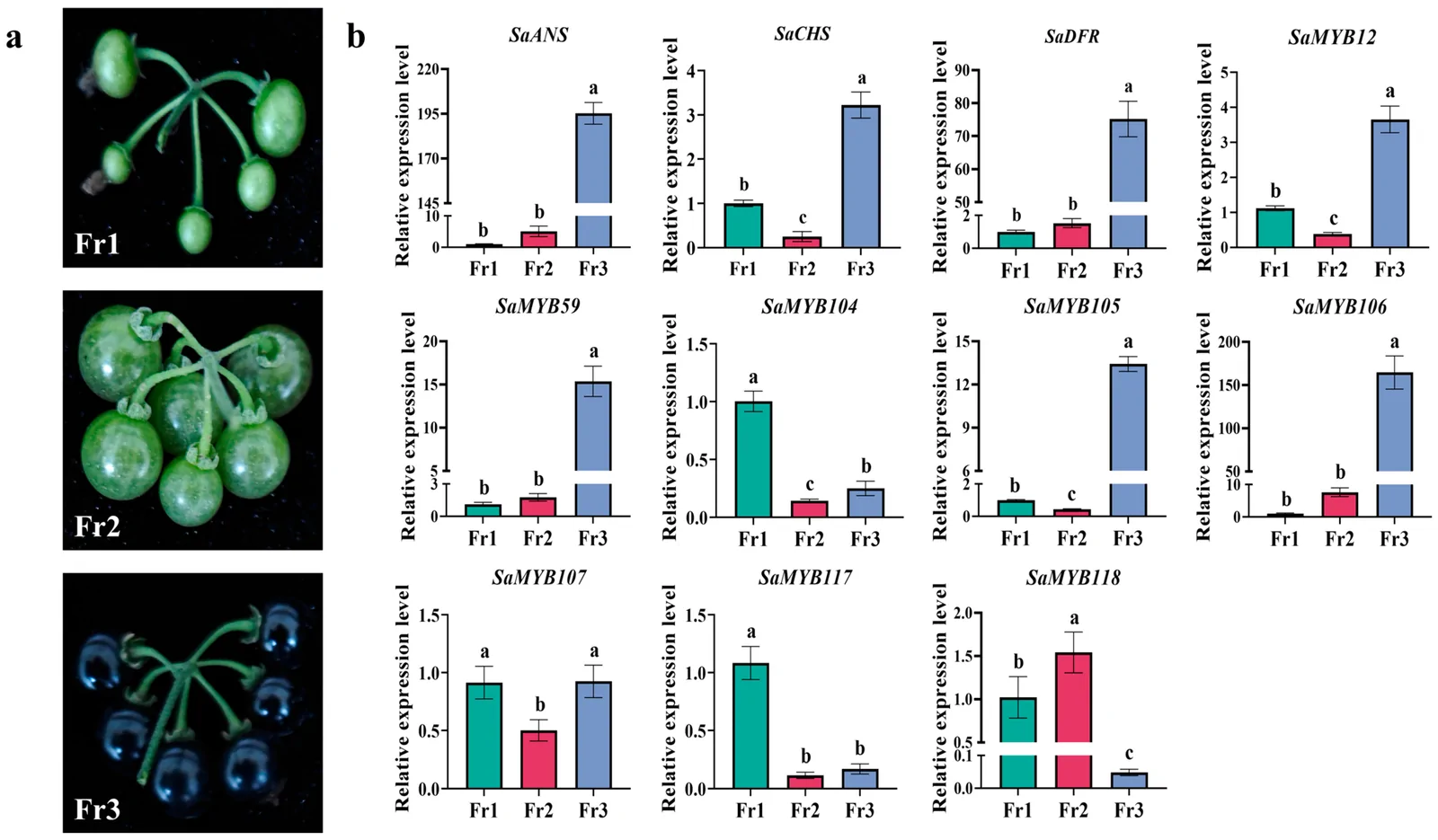
Expression patterns of SaMYB genes during fruit coloration. (**a**) Representative fruits at different coloration stages selected for expression analysis. (**b**) Relative expression levels of structural genes and SaMYB genes. Different lowercase letters indicate significant differences among groups as determined by one-way analysis of variance (ANOVA) followed by Tukey’s multiple comparison test (*p* < 0.05).

The expression patterns of candidate SaMYB genes and anthocyanin biosynthetic structural genes were analyzed by qRT-PCR in fruits at different developmental stages of *S. americanum* (Figure 5a,b). The expression levels of the key anthocyanin biosynthetic structural genes *DFR* and *ANS* gradually increased during fruit development and color transition, reaching their highest levels at the full-purple stage. Compared with the green stage, *ANS* and *DFR* expression increased by approximately 200-fold and 75-fold, respectively, consistent with their association with fruit coloration. The SaMYB genes showed distinct expression patterns during fruit development. Among the S6 subgroup members, *SaMYB106* expression progressively increased during fruit coloration. Its expression increased by approximately 6–9-fold at the color-transition stage compared with the green stage and further increased to more than 100-fold at the full-purple stage, showing the most pronounced induction pattern. *SaMYB105* was markedly upregulated only at the full-purple stage, whereas *SaMYB107* showed little change in expression throughout fruit development. The S4 subgroup members did not show expression patterns consistent with fruit coloration. *SaMYB104* expression markedly decreased after the color-transition stage, whereas *SaMYB12* decreased at the color-transition stage and increased again at the full-purple stage. In the S7 subgroup, *SaMYB59* expression gradually increased during fruit development, whereas *SaMYB117* showed a continuous decrease. The expression level of the S5 subgroup member *SaMYB118* was significantly reduced at the full-purple stage. The expression patterns of *SaMYB59* and *SaMYB106* were consistent with those of the anthocyanin biosynthetic structural genes and the fruit coloration process. Among the candidate genes, *SaMYB106* showed the strongest induction during fruit coloration, whereas *SaMYB59* was the only S7 subgroup member exhibiting a continuous increase in expression throughout fruit development. Therefore, these two genes were selected as representative candidates from different MYB subgroups for further functional validation.

### 3.6. Virus-Induced Gene Silencing of SaMYB59 and SaMYB106

**Figure 6:**
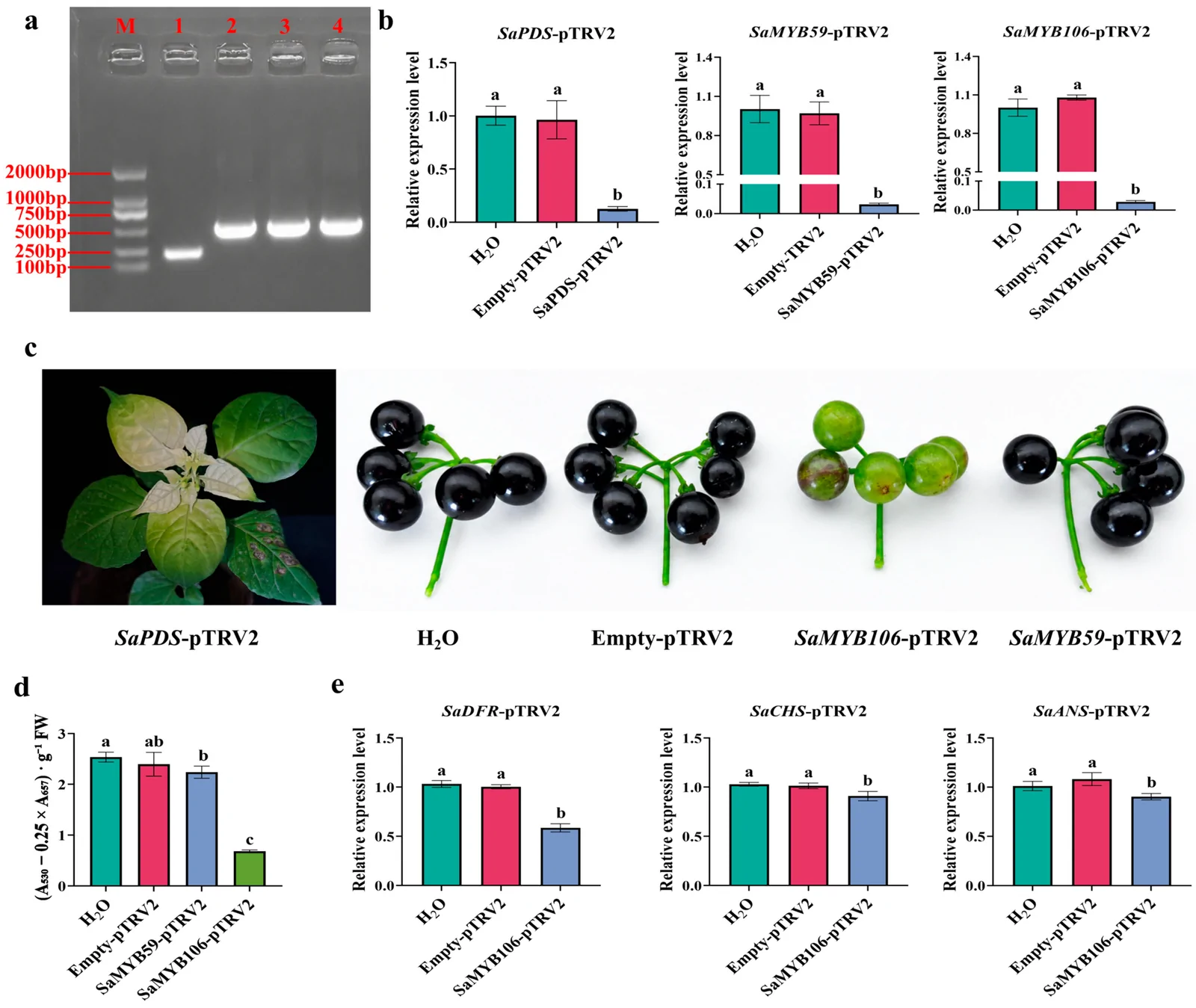
Roles of *SaMYB59* and *SaMYB106* in anthocyanin biosynthesis. (**a**) PCR amplification for the detection of tobacco rattle virus (TRV) in different treatment groups. M, DNA marker; 1, Empty-pTRV2; 2, *SaPDS*-pTRV2; 3, *SaMYB59*-pTRV2; 4, *SaMYB106*-pTRV2. (**b**) Relative expression levels of the target genes in different gene-silencing treatment groups. (**c**) Phenotypes of different treatment groups. (**d**) Relative anthocyanin levels in different treatment groups. (**e**) Relative expression levels of anthocyanin biosynthetic structural genes following *SaMYB106* silencing. Different lowercase letters indicate significant differences among groups as determined by one-way ANOVA followed by Tukey’s multiple comparison test (*p* < 0.05).

To further investigate the potential roles of *SaMYB59* and *SaMYB106* in fruit anthocyanin biosynthesis, virus-induced gene silencing (VIGS) was performed for both genes. Target fragments were selected using the SGN VIGS Tool, which predicted no obvious off-target effects for either construct. PCR analysis of newly emerged leaves from the upper parts of the plants showed that pTRV2 vector-specific amplification fragments were detected in all treatment groups except the H_2_O injection group, indicating that the viral vector had spread to newly emerged tissues. The amplification fragments from the *SaPDS*-pTRV2, *SaMYB59*-pTRV2, and *SaMYB106*-pTRV2 groups were approximately 300 bp longer than that from the Empty-pTRV2 group, indicating that the viral vectors carrying the target silencing fragments had spread to the newly emerged tissues (Figure 6a). qRT-PCR analysis showed that VIGS significantly reduced the transcript levels of the respective target genes. Compared with the Empty-pTRV2 group, the transcript levels of *SaMYB106* and *SaMYB59* were reduced by approximately 97.3% and 96.8%, respectively (Figure 6b). The *SaPDS*-pTRV2 treatment produced an obvious photobleaching phenotype, confirming successful PDS silencing in *S. americanum* and supporting the applicability of the VIGS system for gene silencing in this species (Figure 6c). Among the treatment groups, *SaMYB106* silencing markedly inhibited fruit coloration, whereas no obvious alteration in fruit coloration was observed in *SaMYB59*-silenced plants. Measurement of anthocyanin content in mature fruits at the same developmental stage showed that anthocyanin content was significantly reduced in *SaMYB106*-silenced plants, supporting a positive role for *SaMYB106* in anthocyanin biosynthesis (Figure 6d). To further examine the potential role of *SaMYB106* in anthocyanin biosynthesis, the expression of anthocyanin biosynthetic structural genes was analyzed by qRT-PCR in *SaMYB106*-silenced plants. *DFR* expression was significantly reduced following *SaMYB106* silencing (Figure 6e), suggesting that *SaMYB106* may act upstream of *DFR* and contribute to anthocyanin biosynthesis in *S. americanum* fruits.

## 4. Discussion

The R2R3-MYB transcription factor family is widely involved in plant growth and development, secondary metabolism, and stress responses, and represents one of the major transcription factor families controlling important plant traits [35,42]. During plant evolution, gene duplication has contributed to the expansion of the R2R3-MYB family, followed by functional diversification among duplicated members, resulting in subgroups with distinct regulatory characteristics [43,44]. R2R3-MYB family analyses have been conducted in numerous plants, primarily in cultivated crops and model species [35]. By contrast, systematic characterization of this family in wild *Solanum* species remains limited. In this study, the R2R3-MYB family identified in *S. americanum* exhibited conserved evolutionary relationships with *A. thaliana* R2R3-MYBs, as many SaMYB members clustered within the same subgroups as their *A. thaliana* counterparts. Similar subgroup conservation has been reported in other *Solanum* species, including tomato (*S. lycopersicum*) and potato (*S. tuberosum*), suggesting that R2R3-MYB members may have maintained relatively stable functional diversification patterns within the *Solanum* lineage [45,46]. Gene duplication is considered a major evolutionary force driving the expansion of plant gene families. In *S. americanum*, the presence of multiple syntenic relationships among SaMYB members and Ka/Ks values below 1 for all analyzed syntenic gene pairs suggest that the duplicated SaMYB genes are subject to functional constraints. Some SaMYB genes showed syntenic relationships with multiple SaMYB genes, consistent with the whole-genome triplication event reported in the *Solanum* lineage [47]. Similar duplication-associated expansion patterns have been reported for R2R3-MYB families in various plant species [43,48]. However, expansion of a gene family does not necessarily result in functional redundancy among members. Instead, differences in gene structure and expression patterns may contribute to functional diversification among different subgroups [41,49]. Several SaMYB members were assigned to the S4, S5, S6, and S7 subgroups associated with anthocyanin regulation [18,27,40]. The conservation of anthocyanin-associated MYB subgroups in *S. americanum* provides a basis for identifying candidate genes involved in fruit coloration.

The regulation of anthocyanin biosynthesis represents an important example of functional diversification within the R2R3-MYB family. Members from different MYB subgroups generally exhibit conserved regulatory roles in this process [35,40]. In *A. thaliana*, *AtMYB75*/*PAP1* and *AtMYB90*/*PAP2* activate the expression of anthocyanin biosynthesis-related genes, whereas *AtMYB4* negatively regulates this pathway by repressing genes involved in phenylpropanoid metabolism [18,38,39]. Similar regulatory patterns have been reported in fruit crops, such as *MdMYB10* in apple (*M*. *domestica*) and *VvMYBA* in grapevine (*V*. *vinifera*), which promote fruit coloration through regulation of anthocyanin structural genes [27,50]. In this study, several SaMYB members were assigned to the S4, S6, and S7 subgroups, which showed differences in motif composition, gene structure, and promoter cis-elements. During fruit development, *SaMYB106* and *SaMYB59* exhibited expression patterns associated with anthocyanin accumulation. Notably, *SaMYB106* expression increased markedly during fruit coloration and was consistent with the expression patterns of *DFR* and *ANS*. These results identified *SaMYB106* and *SaMYB59* as candidate regulators of fruit coloration for further functional validation.

R2R3-MYB transcription factors are major regulators within the anthocyanin biosynthetic network. By binding to the promoters of structural genes and modulating their transcription, these factors contribute to anthocyanin accumulation [50,51,52]. In dicot plants, anthocyanin-related MYB proteins generally interact with bHLH and WD40 proteins to form MBW complexes, which activate the expression of late biosynthetic genes, including *DFR*, *ANS*, and *UFGT*, thereby promoting anthocyanin biosynthesis [18,53,54,55]. Therefore, expression profiling alone is insufficient to define the regulatory roles of MYB members, and functional validation is required to determine their positions within the anthocyanin regulatory network [35,52]. In this study, VIGS-mediated suppression of *SaMYB106* significantly reduced purple pigmentation and anthocyanin accumulation in *S. americanum* fruits, indicating that *SaMYB106* is a candidate positive regulator of anthocyanin accumulation during fruit coloration. The transcript level of *SaMYB106* was reduced by approximately 97.3% compared with the Empty-pTRV2 control. Considering that MYB genes within the same subgroup may share sequence similarity, potential off-target effects cannot be completely excluded. However, the silencing fragment was designed using the SGN VIGS Tool, which predicted no obvious off-target sequences, supporting the specificity of *SaMYB106* silencing in this study. In contrast, although *SaMYB59* expression was effectively suppressed by VIGS, no obvious fruit coloration phenotype was observed. This result suggests that *SaMYB59* may have a limited contribution to visible anthocyanin accumulation under the tested conditions. However, its potential regulatory function cannot be excluded and requires further investigation. The function of *SaMYB106* is consistent with the reported roles of S6 subgroup MYB members in other plant species. Similar MYB-mediated regulation has also been reported in Solanaceae crops. Functional studies of *SlAN2*-*like* in tomato (*S*. *lycopersicum*), *SmMYB1* in eggplant (*S. melongena*), and *CaMYB* in pepper (*C. annuum*) have demonstrated that MYB transcription factors contribute to anthocyanin formation in Solanaceae fruits [22,56,57]. Thus, this study not only provides the first systematic characterization of the R2R3-MYB family associated with fruit coloration in *S. americanum* but also identifies *SaMYB106* as a candidate regulator of fruit coloration through expression analysis and VIGS-based functional validation, thereby expanding the functional evidence for S6-type MYB members in wild *Solanum* species. *DFR* is considered an important regulatory enzyme connecting dihydroflavonol conversion with anthocyanin formation, and its expression level is closely associated with final pigment accumulation [58,59]. Several MYB activators promote anthocyanin biosynthesis by inducing *DFR* expression [18,27,50]. Consistently, *SaMYB106* silencing reduced *DFR* expression together with anthocyanin content in *S. americanum* fruits, suggesting that *SaMYB106* may contribute to anthocyanin accumulation through regulation of anthocyanin biosynthetic gene expression.

Anthocyanins not only determine plant tissue coloration but also function as important secondary metabolites involved in stress adaptation and nutritional quality improvement. Therefore, anthocyanin accumulation has become an important target for fruit and vegetable quality improvement [51]. As an important wild resource of Solanaceae, *S. americanum* contains abundant secondary metabolites and exhibits strong environmental adaptability. The pronounced anthocyanin accumulation during fruit maturation makes this species a suitable material for investigating the regulation of fruit coloration in *Solanum* plants. In this study, *SaMYB106* was identified and functionally validated as a positive regulator of anthocyanin accumulation in *S. americanum* fruits. This finding extends the functional understanding of S6-type R2R3-MYB members in wild *Solanum* species and supports the conservation of MYB-mediated regulation of fruit coloration in Solanaceae. MYB transcription factors have been widely studied in metabolic engineering and crop quality improvement because of their regulatory roles in anthocyanin biosynthesis [27,50]. Therefore, *SaMYB106* may serve as a candidate gene for improving fruit pigmentation and anthocyanin-related traits in *Solanum* crops. However, its downstream regulatory network remains unclear. Future studies should identify its direct target genes using yeast one-hybrid, dual-luciferase reporter, and ChIP-qPCR assays and determine its potential involvement in MBW complex formation using yeast two-hybrid, BiFC, or other protein interaction assays. Stable transformation or overexpression studies will further clarify its effects on fruit coloration, anthocyanin composition, and related metabolic pathways.

## 5. Conclusions

In this study, we systematically identified and characterized the R2R3-MYB transcription factor family in *S. americanum* and identified 122 members. Synteny analysis indicated that syntenic SaMYB genes may be subject to functional constraints. Phylogenetic analysis revealed that several SaMYB members clustered within subgroups potentially associated with fruit coloration. Based on phylogenetic relationships, protein structures, promoter cis-elements, and fruit developmental expression patterns, *SaMYB59* and *SaMYB106* were identified as candidate genes potentially involved in anthocyanin regulation. Virus-induced gene silencing further demonstrated that reduced *SaMYB106* expression inhibited purple coloration and decreased anthocyanin accumulation in *S. americanum* fruits, supporting its positive regulatory role in fruit coloration. Moreover, *SaMYB106* silencing reduced *DFR* expression, suggesting that *SaMYB106* may influence anthocyanin accumulation through the regulation of structural genes in the anthocyanin biosynthetic pathway. Collectively, this study highlights the potential role of the *S. americanum* R2R3-MYB family in fruit anthocyanin formation and identifies *SaMYB106* as a candidate regulator of fruit coloration, providing genetic resources for further studies on fruit color formation and the utilization of wild *Solanum* germplasm.

## Data Availability

All data analysed during this study are included in this article.

## Supplementary Materials

The following supporting information can be downloaded at https://www.mdpi.com/article/10.3390/biology15171480/s1, Table S1: Renaming information of SaMYB genes in *S*. *americanum*; Table S2: Primer information; Table S3: Physicochemical properties of SaMYB proteins; Table S4: Syntenic gene pairs and Ka/Ks values of SaMYB genes; Supplementary File S1: Newick file of the SaMYB gene family phylogenetic tree.
